# Effects of phosalone plant pesticide on sperm parameters and sexual hormone levels in Wistar rats: An experimental study

**DOI:** 10.18502/ijrm.v13i9.7683

**Published:** 2020-09-20

**Authors:** Fahime Mazaheri, Kazem Kamali Aliabad, Seyed Mehdi Kalantar, Noosha Ziya, Arezoo Khoradmehr, Morteza Anvari

**Affiliations:** ^1^Department of Biochemistry, Islamic Azad University, Shahrekord Branch, Shahrekord, Iran.; ^2^Research and Clinical Center for Infertility, Yazd Reproductive Sciences Institute, Shahid Sadoughi University of Medical Sciences, Yazd, Iran.; ^3^Department of Arid land and Desert Management, Faculty of Natural Resources, Yazd University, Yazd, Iran.; ^4^The Persian Gulf Marine Biotechnology Research Center, the Persian Gulf Biomedical Sciences Research Institute Bushehr University of Medical Sciences, Bushehr, Iran.

**Keywords:** Organophosphate, Pesticides, Phosalone, Sperm, Sex hormones

## Abstract

**Background:**

Phosalone is an organophosphate insecticide, applied to control of plant pests. This compound has various side effects because it acts as an acetyl cholinesterase enzyme inhibitor.

**Objective:**

To investigate the effects of phosalone on the sperm parameters of and levels of sex hormones in adult male rats.

**Materials and Methods:**

In this experimental study, 16 adult (8-12 wk) male Wister rates (weighing 220-280 gr) were randomly assigned into 4 groups (n = 4/each). Group 1 (control) received only routine adequate water and food; Group 2, 3, and 4 received different low doses of phosalone (60, 90, and 120 mg/kg respectively). The rats were weighed and anesthetized after 48 days. Sperm parameters including number, motility, and viability as well as sex hormones (such as Luteinizing Hormone, Follicle Stimulating Hormone, and testosterone) were evaluated and compared after removing the epididymis tail.

**Results:**

Our results showed that phosalone decreased sperm motility, viability, and number in a dose-dependent manner. The level of FSH and LH was increased, and testosterone was decreased. Also, depending on the dose, phosalone decrease sperm motility and viability (p ≤ 0.001), while the level of FSH and LH was increased and testosterone was decreased (p = 0.861).

**Conclusion:**

Phosalone has negative effects on reproductive indices in male rats and can cause serious damage and decrease the number and sperms motility. It can also cause infertility due to changing the concentration of hormones.

## 1. Introduction

Recent advance in industries and biotechnology has led to creation of products improving the living conditions of humans. However, along with these advances, a large number of physical, chemical, and biotechnical agents have been created that affect the environment. Based on the sensitivity of reproduction, these agents have caused considerable damage to the female and male reproductive system. In the past 50 yr, human exposure to environmental and occupational chemicals has considerably increased, and pesticides have the greatest share among these chemicals (1-3). Pesticides infiltrate the blood circulation and directly lead to the production of infertile sperms by damaging and destroying the cells of the testes tissue or as mutagenic substances in cells (4). Some pesticides are agents disrupting the endocrine glands, causing hormonal imbalance and disruptions in male fertility. The synthesis, storage, release, and bonding of hormones are affected by them, leading to a change in the normal level of reproductive hormones (5, 6). They disrupt the normal functioning of reproductive hormones and indirectly decrease sperm production or lead to production of sperms with abnormal morphology by disturbing hormonal paths in cell differentiation and spermatogenesis (7, 8).

Nithya and coworkers induced reproductive toxicity using lindane pesticide and examined the level of male sex hormones, antioxidants, and histopathology of testes in rats. In this study, 10 healthy rats were treated with 5 mg/kg of lindane for 30 days. The level of male hormones and antioxidants was reduced in the group receiving lindane compared to the control group. Histopathological examination of the rats' testes showed fission in seminiferous tubules, separation of germ cells from the base membrane, and abnormal intracellular space between seminiferous tubules (1, 9). Researchers treated a number of adult rats using gavage with the dose of 0, 33, 54, 75, and 108 mg/kg over 60 days using malathion pesticide, and evaluated the histological, biochemical, and serological alterations. In this study, malathion was reported to decrease the sperm number and motility, body and testicular weight, and mild and severe alterations in seminiferous tubules in rats. They observed that malathion at the dose of 54 mg/kg increased the level of apoptosis in sperm cells, also significantly changing the activity of testicular enzymes by reducing reproductive hormones, for example, FSH and LH. These results indicate that malathion can have destructive effects on rats' reproductive system (10, 11).

Kamura and colleaguesconducted a study to investigate whether dichloro-dimethyl phosphate had diagnosable undesirable effects on the testes of male rats (12). Animals were treated with oral dichloro-dimethyl phosphate at a dose of 20 mg/kg for 45 days. Their body and sex organ weights were significantly decreased. Also, a significant reduction was observed in the sperm density along with 80% infertility in the treated animals. In this study, the effects of phosalone (Zolone) pesticide as a common insecticide in agriculture, on male fertility were investigated. Phosalone is a highly consumed pesticide from the organophosphorus family. It is widely used in farming for its good performance and no negative effect on pollinators, especially honey bees. The mechanism of this toxin is such that it inhibits cholinesterase enzyme in the nervous system of insects functioning as a neurotransmitter in the inter-synaptic space and stimulates the next axon. Phosalone accumulates at the synapse in the body of the insect and consequently continues to stimulate the next nerve since the cholinesterase enzyme required for the degradation of acetylcholine in the synaptic space and elimination of this continuous stimulation by phosalone is blocked (13).

Due to the excessive consumption of pesticides in the agriculture of the country and since there has been very little study on the effects of phosalone on male infertility, this study aimed to investigate the effect of phosalone as a widely used pesticide on sperm parameters and sex hormones of male rats.

## 2. Materials and Methods

In this experimental study, 16 adult male Wister rats (8-12 wk) weighing 220-280 gr was randomly assigned into four groups (n = 4/each) as follows:

• Group 1 (control): only distilled water and usual feed for 48 days

• Group 2 (low dose): usual feed + 60 mg/kg phosalone

• Group 3 (medium dose): usual feed + 90 mg/kg phosalone

• Group 4 (high dose): usual feed + 120 mg/kg of phosalone

Phosalone was given to rats orally trough gavage

Animals were kept in standard conditions, with the optimal temperature of 22-24°C, in clean cages. They were constantly fed on special rat feed and water. After 48 days, equal to a period of spermatogenesis in rats, the rats in all groups were anesthetized using ether and their left testicle was removed by cutting the scrotum. Then, the tail of epididymis was separated by scissors and transferred to 1000 μl of the Ham'sF10 culture medium inside a central Petri dish.

To remove the sperms from the tail of epididymis in the removed tissue, some cuts were gently created using an insulin syringe. Petri dishes containing the prepared suspension were kept in the incubator for 30 min at 37°C and CO${}_{2}$ pressure of 5%. Then, the samples were removed from the incubator and sperm parameters such as number, motility, and viability were studied.

### Analysis of sperm count and motility

“Talebi and colleagues to determine the level of motility of sperms, 48 days later, rats were sacrificed and the caudal epididymis was taken out. It was the placed in 1 mL of culture medium Hams'F10, which was previously placed in the CO${}_{2}$-bearing incubator. Sperms were extracted by slowly tearing up the tissue, and placed in an incubator with 5% CO${}_{2}$ at 37°C for 30 min. Sperms viability was evaluated using the stained eosin-negrosine, and the motility and sperm concentration were evaluated via Makler chamber with ×100 magnification optical microscopes (Olympus Co. Tokyo, Japan). The motility was further classified into progressive, nonprogressive, and immotile" (14).

To examine the sperm count and motility, 10 µl of each sample was placed at the center of the Makler chamber. After placing the lid, the samples were examined with a light microscope at a magnification of ×200. To this end, on each slide, 100 sperms were counted, and sperm number and motility were determined based on the World Health Organization's guidelines (15). Further, to the sperm viability, the eosin-nigrosine staining was performed. To perform this test, 10 µL of each sample was taken by a sampler and smeared on a clean slide. Then, 10 µL of the eosin-nigrosine solution was added to it. With pipetting, the sample and dye were mixed and covered with a cover slip, and viewed under a light microscope at a magnification of ×100. Finally, by counting 100 sperms and determining the number of unstained (viable) and red sperms (dead), the viability of each sample was evaluated.

### Hormone measurement 

After 48 days of treatment with phosalone and anesthetizing the groups, the blood samples were taken from the rats' hearts. Then, the blood in the syringe containing heparin (anticoagulant) was quickly transferred to small tubes. Thereafter, the blood-containing tubes were placed in the centrifuge device for 10 min at 5000 rpm. Next, the tubes were removed from the device; blood serum which had formed a separate phase on the coagulated part was carefully removed by a sampler, poured into normal tubes, and kept at -70°C until hormone measurement. To evaluate the level of sex hormones including FSH and LH, the ELISA technique was applied.

### Ethical consideration

This study was approved by Ethics Committee of the Yazd University, Yazd, Iran (IR.YAZD.REC.1398.007).

### Statistical analysis

Data were analyzed using the Statistical Package for the Social Sciences, version 13.0, SPSS Inc, Chicago, Illinois, USA (SPSS). The differences were compared for statistical significance by one-way ANOVA test. P-value < 0.05 was considered as significant.

## 3. Results

**Figure 1:**
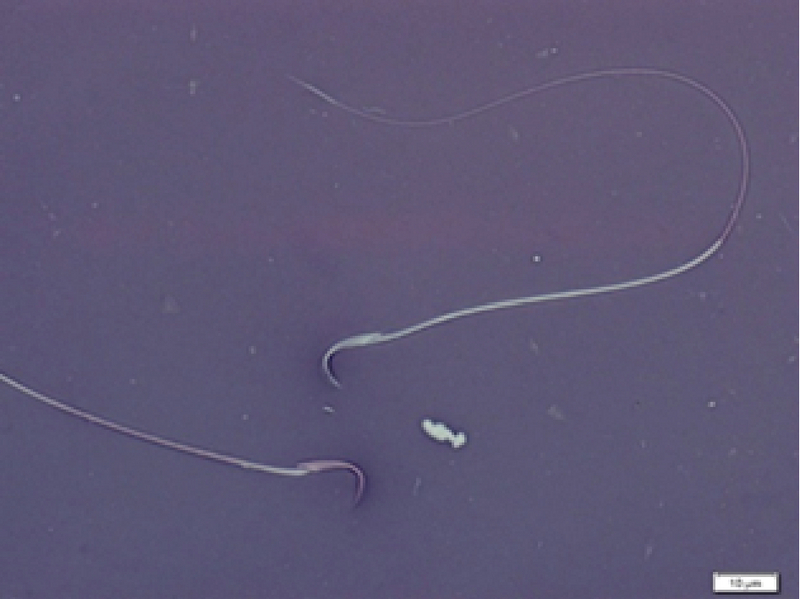
Image of sperms after eosin-nigrosine staining; viable (unstained) and dead (pale violet) sperms (Olympus Microscope. Magnification ×100).

Our results showed that, testes and epididymis had a normal appearance in control group, while they looked smaller in groups 2 and 3. In the groups treated with phosalone, an increase in the dose was reported to reduce the body, testicle, and epididymis weights; this reduction was considerable in the group receiving the high dose compared to the control group. Results showed a significant difference (p < 0.001) between the groups in terms of body, testicle, and epididymis weights (Table I). The results of examination of sperm density and viability in the tail of epididymis showed that these factors were significantly reduced in a dose-dependent manner in the groups receiving phosalone compared to the control group (p < 0.001). In addition, results indicated that the progressive motility of the sperms was decreased upon increasing the dose of phosalone, whereas the slow or nonprogressive motility and the number of immotile sperms were considerably increased (Table II). As shown in Table II, the number of viable sperms was decreased and that of the dead sperms was remarkably increased in a dose-dependent manner in the rats receiving phosalone. The increase in the number of dead sperms in the groups 2 and 3 (medium and high doses, respectively) was considerable compared to the control group (p < 0.001; Figure 1).

### Hormone measurement

The level of serum testosterone was significantly reduced in the groups receiving phosalone compared to the control group in a dose-dependent manner. LH and FSH were also measured in the blood serum of all groups. The level of these hormones was increased in the groups treated with phosalone compared to the control group, however, these differences were not significant (Table III).

**Table 1:** Comparison of the body, testicle, and epididymis weights in four study groups


**Parameters**	**Control group**	**Group 1**	**Group 2**	**Group 3**	**P- value**
**Body weight (gr)**	324.75 ± 4.27	282.25 ±16.70	291.50 ±16.60	262.00 ± 4.69	< 0.001
**Testis weight (gr)**	1.64 ± 0.05	1.46 ± 0.11	1.57 ± 0.13	1.32 ± 0.029	0.002
**Epididymis weight (gr)**	0.36 ± 0.02	0.24 ± 0.018	0.24 ± 0.029	0.18 ± 0.21	< 0.001
Data presented as ANOVA test					

**Table 2:** Comparison of the density, motility, and viability of sperms in the study groups


**Sperm parameters**	**Control group**	**Group 1 **	**Group 2 **	**Group 3 **	**P-value**
**Number of sperm (1×10${}^{6}$)**	66.25 ± 1.70	44.50 ± 7.18	38.00 ± 4.39	22.00 ±2.16	< 0.001
**Live sperm (%)**	88.00± 3.36	66.25 ± 11.5	52.00± 9.41	38.50 ± 5.19	< 0.001
**Dead sperm (%)**	12.00 ± 3.36	33.75 ± 11.58	48.00± 9.41	61.50 ± 5.19	< 0.001
**Progressive motility (%)**	68.50 ± 10.01	48.75 ± 2.98	35.00 ±4.32	21.00 ±1.41	< 0.001
**Nonprogressive motility (%)**	19.75 ± 7.41	27.75± 4.57	33.75 ± 7.41	24.75± 4.71	0.047
**Immotile (%)**	11.75 ± 3.50	23.50 ± 7.14	31.25 ± 10.78	54.25± 4.64	< 0.001
Data are presented as Mean ± SD; ANOVA test					

**Table 3:** Comparison of the testosterone, LH, and FSH levels in the study groups


**Hormones**	**Control group**	**Group 1 **	**Group 2 **	**Group 3 **	**P-value**
**Testosterone**	2.53 ± 0.63	2.09 ± 0.85	1.79± 0.91	1.407 ± 0.89	0.319
**FSH**	1.28 ± 0.67	1.38 ± 1.12	1.62 ± 0.79	1.76 ± 0.85	0.861
**LH**	1.21 ±0.49	1.28 ± 0.72	1.72± 0.75	1.94 ± 0.90	0.466
Data presented as ANOVA test					

## 4. Discussion

The findings of the present study revealed that the oral consumptions of phosalone over 48 days decreased the body, testicle, and epididymis weights in the treated groups compared to the control group. The body weights of the treated animals were significantly lower than those in the control group. Other studies have also reported similar findings. Wissem and colleagues reported that the body weight of animals receiving 250 mg/kg of an organophosphate was decreased, which was confirmed through the present study as well (16). Based on studies worldwide, it is predicted that growing children, especially infants and fetus, should be more sensitive to the toxicity of organophosphates (17). Other studies also support this hypothesis showing that the weight of the fetus is reduced in women exposed to the organophosphates chlorpyrifos (7, 18).

Ambali and co-authors reported that weight reduction following the prescription of organophosphates chlorpyrifos was improved through vitamin E treatment. This finding showed that oxidative stress might be involved in suppressing body weight affected by chlorpyrifos (18). The results of the present study also showed weight reduction in rats. However, Joshi and co-workers indicated that body weight did not change as a result of the effect of organophosphates (19). On the contrary, another study reported that body weight increased via exposure to a type of organophosphates, which was due to the increased fat tissue (18). Reported that many pesticides affect the pituitary, hypothalamus or both, thus reducing the weight of body organ (20). In our study, the decreasing epididymis and testicle weight might be due to the reduction of amounts of male hormones or the anti-androgenic activity of phosalone. Another reason for the decrease in weight of testis can be the reduction in the number of spermatogenic cells and the diameter of seminiferous tubules (20). It seems that by stopping mitosis divisions and inducing cell death, toxins decrease germinal cells, finally leading to a reduction in testis weight. Epididymis weight is mostly dependent on the mass of various spermatogenic cells and a decrease in its weight may be due to the reduction in the volume of tubules as well as number of germinal cells and long spermatids. Moreover, stopping spermatogenesis and inhibiting steroid biosynthesis of Leydig cells might contribute to the reduction in epididymis weight (20). Our results showed a significant reduction in the body, testicle, and epididymis weights in the treatment groups versus controls. These findings confirmed the effectiveness of complications of this pesticide. The use of different doses of phosalone in this study decreased some parameters in the testis. Investigations showed that organophosphate toxins reacted with cell macromolecules and, by increasing peroxidation, induced cell death. It could also postpone mitosis or stop it (20).

Organophosphate toxins alter the activity of antioxidant enzymes that induce reproductive damages, probably by creating free radicals and reactive oxygen species. The phosphorous organic compounds react with the main cell macromolecules such as proteins, nucleic acids, and lipid. Organophosphate causes atrophy in Leydig and Sertoli cells, decreasing the level of blood serum testosterone (21). Tabb and coworkers showed that this pesticide in the long-term decreases spermatogenic, Leydig cells, and testosterone, and increases the LH and FSH (2). Results of the present study showed the same effects on rats' testicle tissue and cells treated with various doses of phosalone compared to the control group over 48 days. Based on our study and other studies (4, 8), it can be concluded that the effects of these toxins depend on their dosage and duration of exposure to them. It seems that phosalone decreases germinal cells by stopping mitosis and inducing cell death. These cells are essential for spermatogenesis, and reduction in them affects cell classes such as spermatocytes, thus leading to a reduction in the number of sperms (20). Various studies represent that organophosphates reduce the level of serum steroid hormones by increasing catabolism, directly affecting testes tissue, and directly and indirectly affecting the endocrine system (22, 23).

In general, numerous factors contribute to the reduction of testosterone, such as inhibiting steroids, a defect in the production of androgens, or degeneration of Leydig cells (2). In this study, the serum level of LH and SFH was increased, but this increase was not significant. Organophosphates increase LH and FSH by preventing the inhibition of return. It seems that phosalone, which has increased these two hormones in this study, belongs to the same group and increases LH and FSH by preventing the inhibition of return. LH plays a significant role in starting and continuing destruction in different stages of spermatogenesis and its increase can reduce testis tissue cells. For the production, maturation, and transfer of sperms, the existence of appropriate levels of sex hormones is essential. A change in the level of testosterone, LH, and FSH can disrupt the process of sperm production (24, 15). Hormonal changes can have various reasons; they may be central or peripheral, that is, sometimes they may be due to a direct damage to pituitary or destruction of the secretion of hormones from the pituitary and hypothalamus. This brain damage may occur in the case of phosalone, although we did not examine the histopathology of brain tissue. Another reason for hormone secretion disruption is the direct tissue damage. This includes reduction in or damage to Leydig cells that secrete testosterone under the effect of LH. In addition, another reason for hormonal changes may be a disorder in hormone metabolism. As LH and FSH are protein hormones with hepatic and renal metabolism, renal and hepatic damage may have caused a disorder. However, testosterone is a lipid hormone with a mostly hepatic metabolism. Therefore, the reason may be the toxicity of hepatic cells (22).

Moreover, Jasuja and co-workers evaluated the effects of the survival of agriculture toxins on the level of health and blood levels of farmers. They found that long-term effects of toxins destroy the tissue of the liver, kidneys, and testis, and lead to a considerable decrease in testosterone level (25). Additionally, another reason for increase in LH is that the toxic inhibits the negative feedback path of LH and, by preventing the inhibition of return, it increases this hormone. Also, the reduction of testosterone increases LH. Simultaneously with the increase in LH, FSH is increased. Epididymis plays an important role in male fertility by providing an optimal liquid medium for the maturation and storage of sperms. The liquid secreted by epididymis is regulated by neurotransmitters that are paracrine and endocrine hormones (26). The physiologic and biochemical integrity of epididymis also depends on the shortage of androgens. Thus, based on the results of the present study, the shortage of androgens may have caused a marked reduction in the diameter of tubules, density of sperm in the tail of epididymis, and a change in its plasma composition (26). A study has shown that, in summer, the level of sperm is decreased among farmers compared to winter, and the reason has been attributed to the use of toxins in summer in a large amount (27).

Patrick and colleagues examined the exposure of organophosphate pesticides and reduction in sperm density, showing that the longer the exposure to this pesticide, the further the reduction in sperm density would be (28). Variation in sperm parameters can be attributed to the direct effect on testicle tissue, leading to a disorder in reproductive function, for example, reduction in density, motility, and morphology of sperms. Specifically, the disorder in strong bonds of Sertoli germ cells leads to a defect in spermatogenesis (29). Deep damage to testis demonstrates the destruction of seminiferous tubules and germinal cells, leading to a disorder in the initial growth of sperm. In addition, exposure to phosalone at low doses affects the functioning of steroid hormones involved in the regulation of fertility processes. Reduction in sperm density and quality is correlated with the reduction in the level of testosterone and oxidative damage, resulting from the inhibition of antioxidant enzyme activity (23). Our findings showed that an exposure to phosalone leads to a disorder in the process of spermatogenesis along with a marked reduction in sperm quality. In general, oxidative damage plays a role in the genotoxic and fertility-related effects under the effect of various metals and organophosphorus pesticides, in line with the results presented here. A body of evidence reports that exposure to arsenic and organophosphate toxins increases ROS in blood and tissues. Increased lipid peroxidation is associated with a change in sperm membrane, reduction in sperm motility, and decrease in fertility potential. Moreover, phosalone can alter the quality of sperm and integrity of sperm DNA and related proteins by producing free radicals in testis, all exacerbating testicle dysfunction. The excessive production of free radicals leads to the probable reduction of mitochondrion membrane along with the reduction in accessible energy which may prevent the motility of sperms (22).

Sperm stimulation is affected by the enzymatic activities of oxidative phosphorylation. The abundance of adenosine triphosphate (ATP) is very important for the normal motility of sperms. Reduction of ATP reduces sperm motility, which may eventually lead to infertility. Reduction in the sperm count in the tail of epididymis is an indicator of the reduction of spermatogenesis as a result of the toxicity of any agent. Biologically speaking, the secretion of gonadotropins is vital for the normal production, growth, and maturation of sperms by testis and epididymis. The decrease in the number of sperms may be due to the change in androgen gonadotropins. Epididymis sperms strongly depend on testosterone and epididymis proteins for their final maturation, development for progressive motility, and fertility capacity. Decrease in sperm motility in the tail of epididymis reflects the low ability of sperms for interaction with the plasma membrane of the ovum. Cyclic nucleotides, especially cyclic adenosine monophosphate (cAMP), are an internal regulator of sperm motility. In addition, it has been reported that sperms develop motility following passage from the epididymis, which may be related to the increase in intracellular cAMP. Phosalone may reduce adenylate cyclase in the epididymis, and as a result, no cAMP is produced, which eventually leads to the reduction in sperm motility (20).

Furthermore, the formation of free radicals can decrease sperm quality. Free radicals can affect the activity of mitochondrion enzymes, leading to a reduction in cell's ability for maintaining the level of their ATP and disorder in the structure of sperm microtubules. Thus, in this way, they disrupt their normal functioning. Slight deprivation of ATP may reduce sperm motility. This mechanism of defect interprets the quality of sperms by decreasing sperm density and motility, and increasing the number of death sperms in the groups treated with phosalone (8, 20). Taib and co-workers reported that the prescription of Fenitrothion (as an organophosphate insecticide) at a low dose in the diet can lead to a slight decrease in the concentration and motility of sperms and morphological changes in them (3). On the other hand, Okamura and colleagues indicated that dichlorvos (an organophosphate) leads to the reduction of the sperm motility percentage without causing any change in the sperm or its morphology (12). This may be due to the inability of dichlorvos in maintaining ATP synthesis in sperm mitochondrion. Moreover, exposure to environmental toxins including organophosphates can cause oxidative stress, which inhibits the activity of antioxidant enzymes and increases lipid peroxidation in the sperm. Organophosphorus pesticides can damage sperm DNA through oxidative stress mechanism. Seminal vesicles play a major role in sperm parameter and hormonal level, and its secretions are necessary for the normal functioning of sperms. As sexual differentiation and growth of sex organs strongly depend on androgens, the reduction in the level of androgen can be a reason for degenerative changes in the tissue structure of seminal vesicle. In a study by Olorunshola and others, a reduction in epithelial complexity and tissue damage to seminal vesicle was reported in the animals treated with chlorpyrifos compared to the control group (29).

## 5. Conclusion 

The results of the present study confirm the previous studies on pesticide phosalone. This toxin depending on the dose has a negative effect on the reproductive indices and changes in the rate of male sex hormones during spermatogenesis. It also causes spermatogenic cell depletion and a decrease in the quality of sperm parameters including decreased sperm count, motility, and viability of them. All of these can cause serious damage to the reproductive system and lead to infertility.

##  Conflict of Interest 

The authors declare that there is no conflict of interest.
